# Physical activity and health-related quality of life in older adults: depression as a mediator

**DOI:** 10.1186/s12877-023-04452-6

**Published:** 2024-01-05

**Authors:** Xiuxiu Li, Pengfei Wang, Yihua Jiang, Yinghua Yang, Feng Wang, Fei Yan, Ming Li, Wenjia Peng, Ying Wang

**Affiliations:** 1 School of Public Health, Fudan University, Shanghai, China; 2 NHC Key Laboratory of Health Technology Assessment, Fudan University, Shanghai, China; 3 Minhang District Mental Health Center of Shanghai, Fudan University, Shanghai, China; 4 Clinical Laboratory Center in Shanghai, Shanghai, China; 5 Shanghai East Hospital, School of Medicine, Tongji University, Shanghai, China

**Keywords:** Depression, Health-related quality of life, Mediator, Older adults

## Abstract

**Background:**

Physical activity(PA) is associated with health-related quality of life (HRQoL) among older adults, and both are associated with mood, such as depression. However, the indirect effects of PA on HRQoL in older adults have not been clearly established. This study explained how different types and intensities of PA were associated with HRQoL while considering the effects of depression in older adults.

**Methods:**

A cross-sectional study was conducted with 7,518 community-dwelling older adults aged 60 years and older. PA (leisure-time, household, and work-related), depression, and HRQoL were measured using the Physical Activity Scale for the Elderly (PASE), the 30-item Geriatric Depression Scale (GDS-30), and the 36-Item Short-Form Health Survey (SF-36), respectively. Information on age, gender, education, monthly income, activities of daily living, smoking, and alcohol drinking was also collected. Regression analysis was used to explore the relationship between PA, depression and HRQoL, and a mediation effect test process was used to verify the mediating mechanism of the depression on this relationship.

**Results:**

The study showed that after adjusting for a set of covariates, SF-36 Physical Component Summary (PCS) scores were negatively associated with depression (B = -2.046, 95% CI [2.584, -1.509]) and positively with PA (p < 0.001). Similarly, SF-36 Mental Component Summary (MCS) scores were negatively associated with depression (B = -11.657, 95% CI [-12.190, -11.124]). In mediation analyses, we found that depression partially mediated the relationship between different types and intensities PA and PCS (moderate leisure-time PA: B = 0.223, 95%CI [0.153,0.293], *P* < 0.001; vigorous leisure-time PA: B = 0.323, 95%CI [0.232,0.413], *P* < 0.001; moderate household PA: B = 0.092, 95%CI [0.045,0.139], *P* < 0.001; vigorous household PA: B = 0.137, 95%CI [0.085,0.190], *P* < 0.001; work-related PA: B = 0.193, 95%CI [0.658,0.190], *P* < 0.001) and MCS (moderate leisure-time PA: B = 1.243, 95%CI [1.008,1.479], *P* < 0.001; vigorous leisure-time PA: B = 1.800, 95%CI [1.585,2.015], *P* < 0.001; moderate household PA: B = 0.496, 95%CI [0.274,0.718], *P* < 0.001; vigorous household PA: B = 0.742, 95%CI [0.521,0.963], *P* < 0.001; work-related PA: B = 1.026, 95%CI [0.819,1.234], *P* < 0.001).

**Conclusions:**

This study suggested that leisure-time, household, and work-related PA were negatively associated with depression, while positively affecting HRQoL in Chinese older adults. The relationships between different types and intensities of PA and HRQoL were mediated by depression. Interventions aimed at promoting purposeful exercise and different types of PA may have mental health benefits. It is recommended that geriatric health managers and healthcare planners prioritize interventions to help improve PA intensities, alleviate depressive symptoms to promote beneficial effects on HRQoL in older adults.

**Supplementary Information:**

The online version contains supplementary material available at 10.1186/s12877-023-04452-6.

## Background

The health benefits of physical activity (PA) are well known, including a reduced risk of non-communicable diseases (NCDs) such as cardiovascular disease, hypertension, diabetes, and others. In addition, PA has a positive impact on mental health and can delay the onset of dementia. However, the duration of PA in modern society is becoming shorter and the trend of a sedentary lifestyle is growing, with a large proportion of people spending a significant part of the day sitting still [1]. An analysis showed that in 2016, more than one-quarter of adults worldwide did not get enough PA, putting more than 1.4 billion adults at risk of developing or exacerbating diseases related to inactivity, a phenomenon that is particularly pronounced among older adults [2]. Numerous epidemiological and laboratory studies have confirmed that PA is not only associated with all-cause mortality, psychiatric and cardiovascular morbidity in older adults, but also leads to fatigue, frailty and cognitive decline, ultimately worsening an individual’s health-related quality of life (HRQoL) [3].

HRQoL is widely used clinically to assess the health effects of PA in older adults because it provides a multidimensional perspective that takes into account the person’s emotional and physical functioning as well as social well-being [4, 5]. Although studies have shown that physical inactivity may adversely affect HRQoL, some issues have not been adequately addressed. First, most of these studies have a small sample size [6, 7] and focus on young and middle-aged people [8, 9]. However, because PA and HRQoL change with age, the results from these studies may not be representative of older adults. In addition, the vast majority of studies focus only on the amount of PA, not the type of PA [10], leading to inconsistent conclusions [6, 11, 12]. Lastly, few studies have explored the mechanisms by which PA leads to a decrease in HRQoL. Therefore, there is a need to confirm the association between PA (type) and HRQoL using a large sample of older adults and to explore the mechanisms behind this association.

Further exploration of the mechanisms provides new insights into how and when independent variables influence dependent variables. In the present study, we considered depression as a mediator in the relationship between PA and HRQoL for the following reasons. First, a study of an Asian older adult population found that PA was associated with depressive symptoms (OR = 1.201, 95% CI [1.035, 1.393]) [13]. A study using a longitudinal design found that heavy and vigorous intensities of PA significantly predicted low depressive symptom scores 4 years later [14]. In addition, studies have shown that depressive symptoms are associated with low HRQoL in older adults [15, 16]. For example, one study found a significant negative association between depression and HRQoL (OR = -1.23, 95% CI [− 1.72, − 0.72]) [16]. Therefore, it is plausible that depression mediates the relationship between PA and HRQoL. A meta-analysis showed that PA improves physical and mental health in depressed patients [17]. A randomized controlled trial of older people with insomnia found that aerobic exercise such as walking, cycling or using a treadmill improved quality of life in older people [16]. The various types of PA may influence HRQoL directly or indirectly through depression as a mediator.

**Fig. 1:**
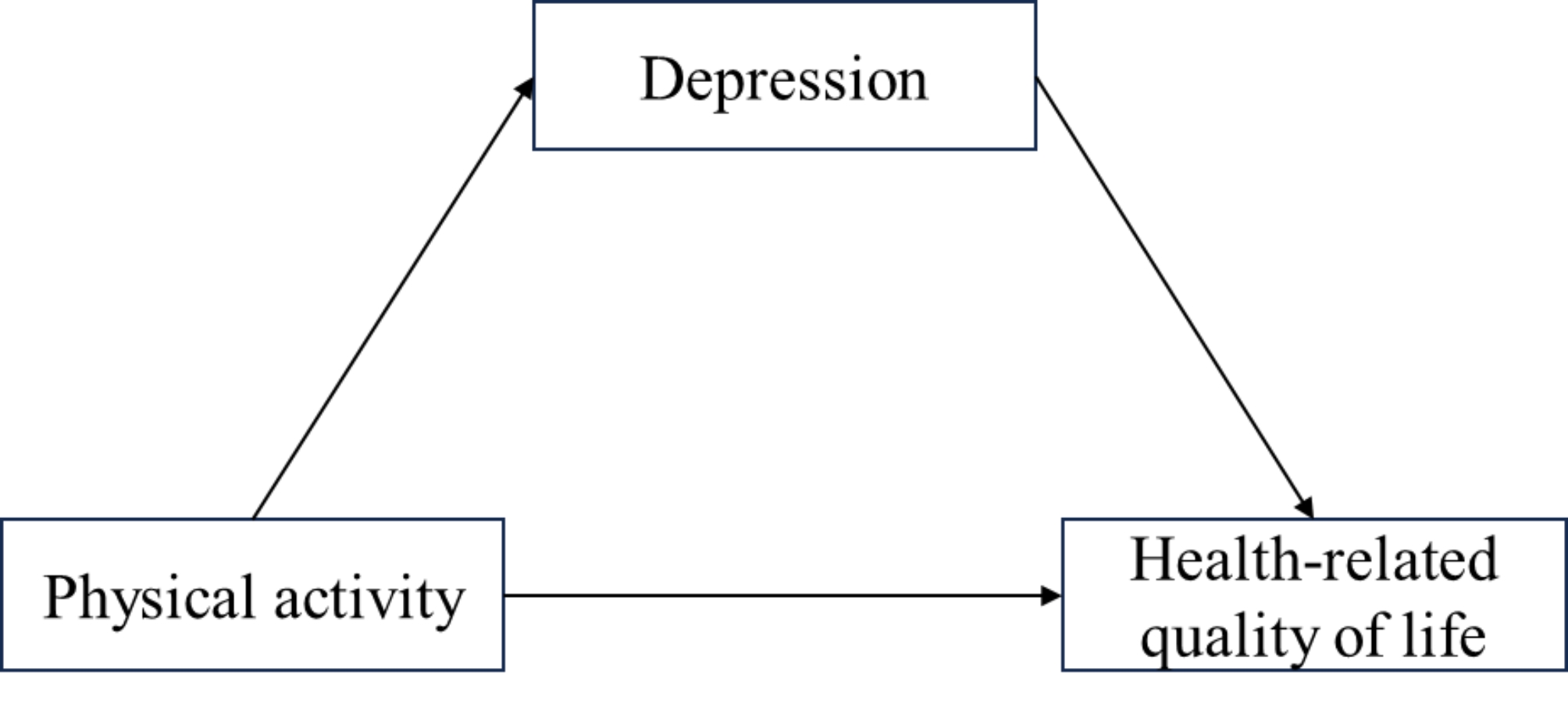
The effect of physical activity on health-related quality of life mediated by depression

It is crucial to validate inferences with a large sample size to determine the relationship between PA and HRQoL and whether depression may be a potential explanation for the relationship between PA and impaired HRQoL. Therefore, the purpose of this study was twofold: (1) to examine the relationship between PA and HRQoL based on data from older adults in the Chinese community, and (2) to explore whether depression mediates between the different types and intensities of PA and HRQoL. Figure 1 illustrates the research model.

## Methods

### Study design

A cross-sectional survey was undertaken in August 2019 in the city of Shanghai. We used a multi-stage random group sampling method (see Supplementary Method). Our selection of older adults was based on the following inclusion criteria: aged 60 and older, able to communicate with the investigator and no significant symptoms of dementia or neurocognitive impairment. Similarly, the exclusion criteria were inability to understand and comply with the study’s assessment protocol or having a severe neurocognitive impairment such as dementia (confirmed by a community physician based on health information). A total of 7,518 older adults were included. Each participant was informed of the purpose of the study and signed an informed consent form. The investigators, all of whom had a medical background and received specialized training, conducted face-to-face surveys with participants at community health centers or neighborhood committees. The questionnaires were reviewed by the researchers for quality control and questionnaires of substandard quality were excluded. This research protocol was approved by the Institutional Review Board Ethics Committee of Fudan University (reference number: IRB#TYSQ 2019-2-03). We obtained written informed consent from participants prior to data collection. And all methods were performed in accordance with the relevant guidelines and regulations.

### Measures

#### Physical activity

The Physical Activity Scale for the Elderly (PASE) was used to assess PA. The PASE asks about participation in 10 activities such as walking, light leisure activities, moderate leisure activities, vigorous leisure activities, sports specifically designed to increase muscle strength and endurance, housework, home maintenance, gardening, yard work, caring for others and paid or volunteer work [18]. In order to compare the relationship between different types of PA and depression and HRQoL, leisure time activities and household activities were classified into respective quartiles of light, moderate and vigorous in this study [19, 20]. The total physical activities groups were divided into light (< 115.75), moderate (115.75-163.25), and vigorous (> 163.25) by the total PASE scores. The leisure-time activities groups were divided into light (< 8.60), moderate (8.60-28.99), and vigorous (> 28.99) depending on the leisure-time activities scores of PASE. The household activities groups were categorized as light (< 86), moderate (86–136), and vigorous (> 136) depending on the household activities scores of PASE. The working-related activities groups were categorized according to that the respondent answered “yes” or “no” to the question “During the past 7 days, did you work for pay or as a volunteer?”.

#### Depressive symptoms

The 30-item Geriatric Depression Scale (GDS-30) was used to assess depressive symptoms. Participants responded to each item with ‘yes’ or ‘no’. Scores can range from 0 to 30, and a threshold of ≥ 11 points was used to define depression [21]. Cronbach’s alpha for the scale was 0.991.

#### Health-related quality of life

HRQoL was assessed using the 36-Item Short-Form Health Survey (SF-36). The SF-36 has 8 subscales divided across physical and psychological domains: Physical Function (PF), Role Physical (RP), Bodily Pain (BP), Global Health (GH), Vitality (V), Social Function (SF), Role Emotional (RE) and Mental Health (MH). Scores range from 0 to 100, with higher scores reflecting greater HRQoL, with a mean score of 50 (SD = 10) in the general US population [22]. The scale can be further aggregated and standardized into two components or scales: the Physical Component Summary (PCS) and the Mental Component Summary (MCS) [23].The PCS is calculated by positively weighting the 4 subscales in the physical domain (PF, RP, BP, GH) and the remaining mental domain subscales negatively. The MCS is calculated by positively weighting the 4 subscales in the mental domain (MH, V, SF, RE) and negatively weighting the 4 physical domain subscales. In this study, the Cronbach’s alpha for the SF-36 was 0.855.

#### Covariates

We collected demographic, lifestyle and health level variables as covariates. The following covariates were included: (1) age in years (60–69 = 1, 70–79 = 2, ≥ 80 = 3); (2) gender (male = 0, female = 1); (3) marital status (married = 0, widowed/divorced/unmarried = 1); (4) education (primary school or lower = 0, middle school or higher = 1); (5) monthly income (≤ 2,000 yuan = 1, 2001–5000 yuan = 2, ≥ 5001 yuan = 3); and (6) living situation (living with spouse or children = 0, living alone = 1). Smoking (7) and alcohol drinking (8) were divided into three categories (non-smoker, former and current; and non-drinker, former and current). Activities of daily living (9) was assessed using the Barthel Index for Activities of Daily Living (BADL) and the Lawton Instrumental Activities of Daily Living (IADL) scale [19]. Basic ADL was assessed using the BADL (score range, 0–100) [20]. For the Lawton IADL scale, the first option selected for each item was rated as 1, and the other options 0. If a question was rated 0, the person was judged to have a disability.

### Statistical analysis

We used the Statistical Package for the Social Sciences (SPSS 25.0) software for the data analysis (IBM, Armonk, NY, USA). Categorical data were calculated as frequencies and percentages. Continuous data were calculated as means and standard deviations (SD). We conducted mediation analyses using Mplus8 to examine the effect of PA on PCS and MCS, considering the mediating effect of depression after controlling a set of covariates. We also conducted a series of regression analyses to assess the statistical significance of the mediation pathway. If the bootstrap confidence interval (CI) for the indirect effect did not include zero, the mediation effect was considered significant.

## Results

### Participants’ characteristics

Table 1 presents the sample’s characteristics. Most participants were aged 60–69 years (58.5%, n = 4,401); 53.7% were women, and most participants (84.5%) were married. The mean PCS and MCS scores were 46.25 (SD = 9.82) and 53.43 (SD = 8.32), respectively. Of the total sample, 12.6% (n = 950) had depression, as defined by a score of 11 or higher. Approximately 40% (n = 2,970) reported participating in mild leisure-related activities and 34.8% (n = 2,615) in light household activities, a higher proportion than for moderate and vigorous intensities. In addition, 89.6% (n = 6,735) had no work-related activities. Their mean IADL and BADL scores were 6.72 and 95.94, respectively.

**Table 1:** Characteristics of the Sample

Characteristic	N = 7518	Characteristic	N = 7518
Age, mean (SD)	70.27 (8.06)	Cigarette smoking, n (%)	
Age group(years), n (%)		Non-smoker	3947 (52.5)
60–69	4401 (58.5)	Former	538 (7.2)
70–79	2055 (27.3)	Current	3033 (40.3)
≥80	1062 (14.1)	Total physical activities, n (%)	
Gender, n (%)		Light (< 115.75)	2507 (33.3)
Male	3484 (46.3)	Moderate (115.75–163.25)	2514 (33.4)
Female	4034 (53.7)	Vigorous (> 163.25)	2497 (33.2)
Education, n (%)		Leisure-time activities, n (%)	
Primary school or lower	3057 (40.7)	Light (< 8.60)	2970 (39.5)
Middle school or higher	4461 (59.3)	Moderate (8.60–28.99)	2045 (27.2)
Marital status, n (%)		Vigorous (> 28.99)	2503 (33.3)
Married	6356 (84.5)	Household activities, n (%)	
Widowed/divorced/never married	1162 (15.5)	Light(< 86)	2615 (34.8)
Monthly income, n (%), Yuan		Moderate (86–136)	2599 (34.6)
≤2000	1497 (19.9)	Vigorous (> 136)	2304 (30.6)
2001–5000	4767 (63.4)	Work-related activities, n (%)	
≥5001	1254 (16.7)	No	6735 (89.6)
Physical function, mean (SD)		Yes	783 (10.4)
Instrumental activities of daily living score	6.72 (2.25)	Depression, n (%)	
Basic activities of daily living score	95.94 (12.15)	No	6568 (87.4)
Alcohol drinking, n (%)		Yes	950 (12.6)
Non-drinker	4112 (54.7)	36-Item Short-Form Health Survey, mean (SD)	
Former	278 (3.7)	Physical Component Summary	46.25 (9.82)
Current	3128 (41.6)	Mental Component Summary	53.43 (8.32)

### Multivariate analyses

**Fig. 2:**
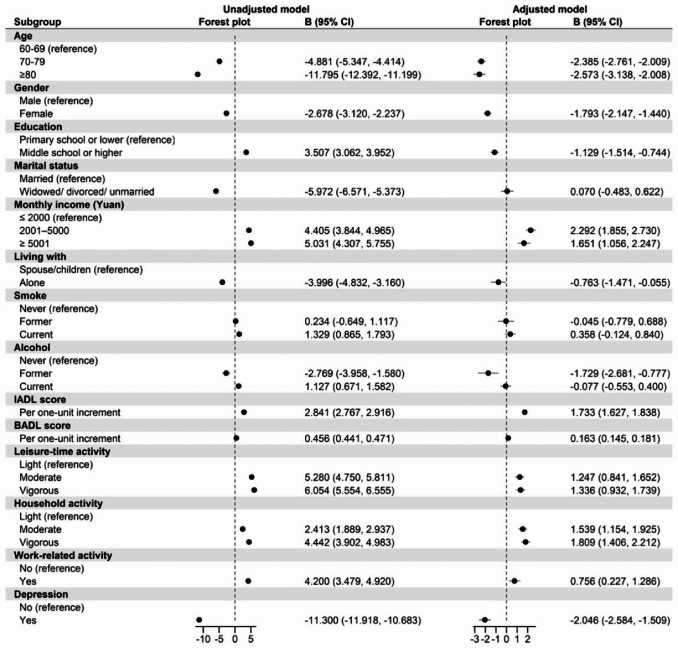
Relationships Between Participants’ Characteristics and Physical Component Summary Score (Univariable and Multivariable Regression)

**Fig. 3:**
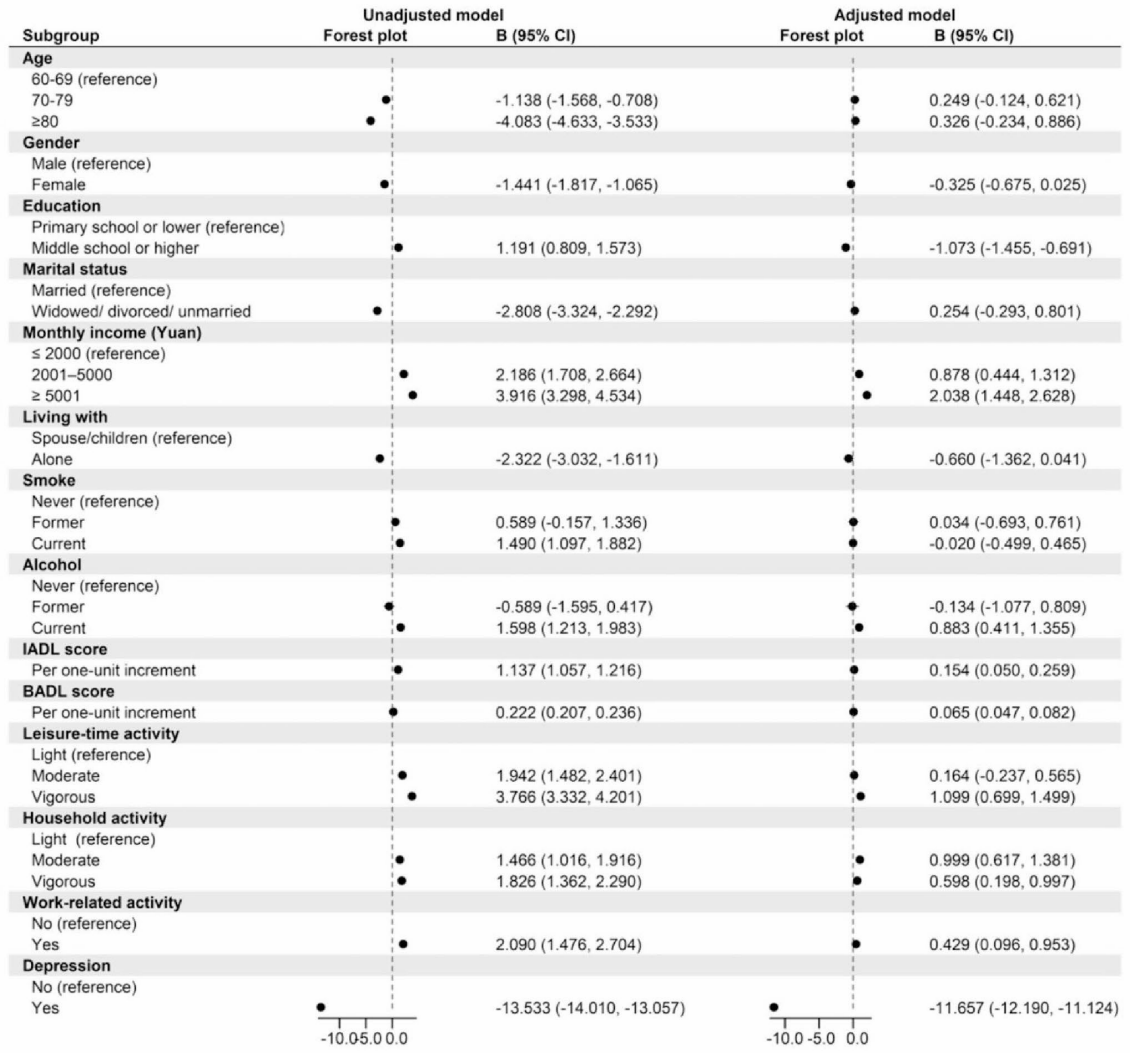
Relationships Between Participants’ Characteristics and Mental Component Summary Score (Univariable and Multivariable Regression)

Figures 2 and 3 show the relationship between the variables related to HRQoL. After adjusting for sociodemographic variables (age, gender, education level, marital status, monthly income, lifestyle, cigarette smoking and alcohol drinking), PCS scores were negatively associated with depression (B = -2.046, 95% CI [-2.584, -1.509]) and positively with the three types of PA (p < 0.001). For leisure-time activities, moderate and vigorous intensities scores were 1.247 (95% CI [0.841, 1.652]) and 1.336 (95% CI [0.932, 1.739]) points higher than the PCS score for light intensity, respectively. Regarding household activities, PCS scores for moderate and vigorous PA were 1.539 (95% CI [1.154, 1.925]) and 1.809 (95% CI [1.406, 2.212]) points higher than those for light PA, respectively. A difference in PCS scores was also found between participants with and without work-related activities; those with work-related activities scored 0.756 (95% CI [0.227, 1.286]) points higher than those without work-related activities. Similarly, MCS scores were negatively associated with depression (B = -11.657, 95% CI [-12.190, -11.124]). In the categories of leisure-related and household activities, moderate and vigorous activity scores were 0.164 (95% CI [-0.237, 0.565]), 1.099 (95% CI [0.699, 1.499]), 0.999 (95% CI [0.617, 1.381]) and 0.598 (95% CI [0.198, 0.997]) points higher than the MCS scores for light-intensity activities, respectively. The MCS score for participating in work-related activities was 0.429 (95% CI [0.096, 0.953]) points higher than the score for no participation in work-related activities.

### Mediation analysis

As shown in Figs. 2 and 3, and Table 2, different types and intensities of PA positively correlated with HRQoL (p < 0.001), and depression negatively correlated with PA and HRQoL (p < 0.001). Depression had a mediating effect on the relationship between PA and HRQoL related to physical health. The proportion of the mediating effect varied across types and intensities; for example, depression had the largest indirect effect of 16.74% (B = 0.323, 95% CI [0.232, 0.413]) for vigorous leisure-time activities and PCS, and the smallest indirect effect of 5.53% (B = 0.092, 95% CI [0.085, 0.190]) for moderate household activities and PCS.

**Table 2:** The Effects of Physical Activity on Quality of Life Through Depression

Variable	PCS	Mediated effect		MCS	Mediated effect	
	Effect	95% CI	(%)	Effect	95% CI	(%)
**Leisure-time activities** (Ref: Light)						
**Moderate**						
Total effect	1.508***	1.097 to 1.920		1.437***	0.973 to 1.901	
Direct effect	1.285***	0.879 to 1.692		0.193	-0.208 to 0.595	
Indirect effect	0.223***	0.153 to 0.293	14.79	1.243***	1.008 to 1.479	86.50
**Vigorous**						
Total effect	1.930***	1.527 to 2.332		2.989***	2.547 to 3.432	
Direct effect	1.607***	1.209 to 2.005		1.190***	0.797 to 1.582	
Indirect effect	0.323***	0.232 to 0.413	16.74	1.800***	1.585 to 2.015	60.22
**Household activities** (Ref: Light)						
**Moderate**						
Total effect	1.664***	1.276 to 2.052		1.516***	1.075 to 1.957	
Direct effect	1.572***	1.186 to 1.959		1.021***	0.639 to 1.402	
Indirect effect	0.092***	0.045 to 0.139	5.53	0.496***	0.274 to 0.718	32.72
**Vigorous**						
Total effect	2.073***	1.669 to 2.476		1.499***	1.046 to 1.953	
Direct effect	1.935***	1.535 to 2.336		0.757***	0.361 to 1.153	
Indirect effect	0.137***	0.085 to 0.190	6.61	0.742***	0.521 to 0.963	49.50
**Work-related activities** (Ref: No)						
Total effect	1.188***	0.133 to 0.254		1.687***	1.129 to 2.245	
Direct effect	0.995***	0.468 to 1.523		0.660***	0.141 to 1.180	
Indirect effect	0.193***	0.658 to 1.718	16.25	1.026***	0.819 to 1.234	60.82

N = 7,518. Models adjusted for age, gender, education, marital status, monthly income, living situation (alone or with spouse/children), smoking status, alcohol intake, instrumental activities of daily living and basic activities of daily living. PCS: Physical Component Summary; MCS: Mental Component Summary; CI: confidence interval. ***p < 0.001

The same trend was evident for HRQoL related to mental health, where depression partially mediated the relationship between moderate leisure-time activities and HRQoL related to mental health. The lowest effect was for moderate household activities and mental health at 32.72% (B = 0.496, 95% CI [0.274, 0.718]).

## Discussion

This is the first study to investigate whether depression mediates the relationship between PA and HRQoL in Chinese community-dwelling older adults.

In this study, PA was negatively associated with depression. In a meta-analysis of 49 prospective cohort studies, including 1,837,794 person-years, individuals with vigorous PA were 17% less likely to develop depression than those with light PA [24]. A similar study conducted with 88,522 adults in Brazil demonstrated that different types of PA reduced the odds of depression through different mechanisms, which was consistent with our findings [25]. The mechanisms studied included neurophysiological hypothalamic–pituitary–adrenal axis regulation, increase in hippocampal volume, and regulation of pro-inflammatory factors. From the socio-psychological perspective, PA may alleviate depression by providing opportunities for social interaction, thereby contributing to self-esteem and self-efficacy [26].

Furthermore, the results indicated that depression was negatively associated with overall HRQoL. This finding is consistent with previous studies [27, 28]. Because depression can include feelings of sadness, helplessness, hopelessness and a generally poor outlook on life, HRQoL will be negatively affected [29].

Our study showed a positive association between PA intensities and HRQoL. Different types of PA of the same intensity had different effects on HRQoL; however, both moderate and vigorous PA yielded better HRQoL than light PA. This is consistent with the large representative national survey from England findings, in which higher intensities of PA are associated with better HRQoL [30]. An increase in PA intensity is associated with improvements in physical self-esteem and positive emotions. An improvement in mood has a direct effect on life satisfaction. Overall, this indicates that the effect of PA on HRQoL is mediated by positive emotions, and the intensity and type of PA are predictors of HRQoL in community-based older adults.

The results of the mediation analysis showed that PA had a significant indirect effect on HRQoL through depression. Several studies have shown that PA is a protective factor against depressive symptoms and that even small amounts of PA reduce the incidence of future depressive episodes [31, 32]. This may result from a range of biochemical and psychosocial factors, including biological mechanisms that increase neurogenesis, reduce inflammation and oxidative markers [33], and activate the endocannabinoid system through PA [34]. In addition, PA may directly increase psychological factors, including reduced stress, tension, and anxiety, and increased self-esteem or perceptions of physical ability. Finally, increased levels of physical fitness lead to subjective and objective improvements in physical health status [32, 35]. This means that we may be able to improve the HRQoL for older adults by increasing PA levels and reducing the incidence of depression. It should be noted that it is likely there are other mediator variables in addition to depression linking PA to HRQoL that were not considered in this study, such as social support [36]. Furthermore, HRQoL can act as a mediator in the relationship between PA and depression, which was found in a study of women with chronic illness [37]. Lastly, a study also showed a partial mediating effect of depression in the relationship between PA and HRQoL, further confirming that PA, depression and HRQOL have a reciprocal relationship [38].

Depression mediated the association between PA and mental HRQoL more than physical HRQoL, but the mechanism remains unclear. According to a longitudinal study on the relationship between PA and HRQoL in older women with a history of depression, higher PA intensities were associated with better mental HRQoL than physical HRQoL [39]. This may be because the relative presence or absence of depression reveals whether older adults have a good sense of self-efficacy [40] and more adequate social support [36].

There are several limitations in this study. First, we used self-report scales for measurement, which may lead to information bias. Second, the study utilized a cross-sectional research design, so we were unable to establish a causal relationship between PA and HRQoL. Despite this limitation, it has some noticeable strengths, including a large sample of 7,518 older adults. Important confounders were included in the analysis, and our results provide evidence that depression is a mediator between PA and HRQoL in older adults.

## Conclusion

This study suggested that leisure-time, household, and work-related PA were negatively associated with depression, while positively associated with HRQoL in older adults. The relationships between different types and intensities of PA and HRQoL were mediated by depression. Interventions aimed at promoting purposeful and different types of PA may improving HRQoL by reducing depression symptoms. It is recommended that geriatric health managers and healthcare planners prioritize interventions to help older adults to take more PA, alleviate depressive symptoms to improve HRQoL.

### Electronic supplementary material

Below is the link to the electronic supplementary material.

Supplementary Material 1

## Data Availability

Our data is presently unavailable and inaccessible to other researchers for replication purposes because the researcher has not completed planned or expected analyses for future publications. This study was not pre-registered in any independent institutional registry. If you would like to have access to the raw data from the study, an application should be made by the corresponding author of this paper, Prof. Ying Wang, email: wangying1013@fudan.edu.cn.

## Abbreviations

PA: Physical activity
HRQoL: Health-related quality of life
PASE: Physical Activity Scale for the Elderly
GDS30: The 30-item Geriatric Depression Scale
BADL: Barthel Index for Activities of Daily Living
IADL: Instrumental Activities of Daily Living
PCS: Physical Component Summary
MCS: Mental Component Summary
