# Supplementary material for: Physical activity and health-related quality of life in older adults: depression as a mediator
Source: BMC Geriatr. 2024 Jan 5;24:26. doi: 10.1186/s12877-023-04452-6 (PMC10770982; doi:10.1186/s12877-023-04452-6)
Supplement: Supplementary file 1 — Supplementary Material 1 [file 12877_2023_4452_MOESM1_ESM.docx]

**Supplementary Method**

***Sampling Method***

We used a three-stage stratified cluster sampling method. In the first stage, all 18 districts in Shanghai were divided into three groups based on their distance from the city center: downtown, inner suburb, and outer suburb. One district was randomly selected from each group—the Hongkou, Minhang, and Qingpu districts. Furthermore, one community each—Jiangwan, Xinzhuang, and Jinze—were selected from the three districts. The samples were randomly selected from the census database of older adults in Hongkou, Minhang, and Qingpu districts. The older adults in the three communities were stratified by age and sex, and one neighborhood each was randomly selected from the 35 neighborhoods in Jiangwan, 53 neighborhoods in Xinzhuang, and 35 neighborhoods in Jinze. If the population in the neighborhood did not meet the needs of the sample, the sampling process continued with the subsequent neighborhood according to random sampling until the number met the requirements. Finally, 2,500, 2,518, and 2,500 older adults were interviewed in the Hongkou, Minhang, and Qingpu districts, respectively, with 7,518 older adults participating in the present study.

**Supplementary Table S1. Correlations between the study variables**

|  | 1 | 2 | 3 | 4 | 5 |
| --- | --- | --- | --- | --- | --- |
| 1. PCS | - |  |  |  |  |
| 2. Depression | 0.448** | - |  |  |  |
| 3. Leisure time | 0.271** | -0.334** | - |  |  |
| 4. Household | 0.220** | -0.159** | 0.244** | - |  |
| 5. Work-related | 0.134** | -0.122** | 0.210** | 0.031** | - |

|  | 1 | 2 | 3 | 4 | 5 |
| --- | --- | --- | --- | --- | --- |
| 1. MCS | - |  |  |  |  |
| 2. Depression | 0.533** | - |  |  |  |
| 3. Leisure time | 0.197** | -0.334** | - |  |  |
| 4. Household | 0.124** | -0.159** | 0.244** | - |  |
| 5. Work-related | 0.063** | -0.122** | 0.210** | 0.031** | - |

** *P*<0.01

**Supplementary Table S2. Characteristic of PCS and MCS (univariable and multivariable regression)**

| **Factors** | **Unadjusted** |  |  | **Adjusted** |  |  |
| --- | --- | --- | --- | --- | --- | --- |
| **Model 1: Dependent variable, PCS scores** | B (95% CI) | β | P | B (95% CI) | β | P |
| Age |  |  |  |  |  |  |
| 60-69 | Reference |  |  | Reference |  |  |
| 70-79 | -4.881 (-5.347, -4.414) | -0.222 | < 0.001 | -2.385 (-2.761, -2.009) | -0.108 | < 0.001 |
| ≥80 | -11.795 (-12.392, -11.199) | -0.418 | < 0.001 | -2.573 (-3.138, -2.008) | -0.091 | < 0.001 |
| Sex |  |  |  |  |  |  |
| Male | Reference |  |  | Reference |  |  |
| Female | -2.678 (-3.120, -2.237) | -0.136 | < 0.001 | -1.793 (-2.147, -1.440) | -0.091 | < 0.001 |
| Education |  |  |  |  |  |  |
| Primary school or lower | Reference |  |  | Reference |  |  |
| Middle school or higher | 3.507 (3.062, 3.952) | 0.175 | < 0.001 | -1.129 (-1.514, -0.744) | -0.056 | < 0.001 |
| Marital status |  |  |  |  |  |  |
| Married | Reference |  |  | Reference |  |  |
| Widowed/ divorced/ unmarried | -5.972 (-6.571, -5.373) | -0.22 | < 0.001 | 0.070 (-0.483, 0.622) | 0.003 | 0.804 |
| Monthly income (Yuan) |  |  |  |  |  |  |
| ≤ 2000 | Reference |  |  | Reference |  |  |
| 2001–5000 | 4.405 (3.844, 4.965) | 0.216 | < 0.001 | 2.292 (1.855, 2.730) | 0.112 | < 0.001 |
| ≥ 5001 | 5.031 (4.307, 5.755) | 0.191 | < 0.001 | 1.651 (1.056, 2.247) | 0.063 | < 0.001 |
| Living situation |  |  |  |  |  |  |
| Living with spouse/children | Reference |  |  | Reference |  |  |
| Living alone | -3.996 (-4.832, -3.160) | -0.107 | < 0.001 | -0.763 (-1.471, -0.055) | -0.021 | 0.035 |
| Smoking |  |  |  |  |  |  |
| Non-smoker | Reference |  |  | Reference |  |  |
| Former | 0.234 (-0.649, 1.117) | 0.006 | 0.603 | -0.045 (-0.779, 0.688) | -0.001 | 0.904 |
| Current | 1.329 (0.865, 1.793) | 0.066 | < 0.001 | 0.358 (-0.124, 0.840) | 0.018 | 0.146 |
| Alcohol drinking |  |  |  |  |  |  |
| Non-drinker | Reference |  |  | Reference |  |  |
| Former | -2.769 (-3.958, -1.580) | -0.053 | < 0.001 | -1.729 (-2.681, -0.777) | -0.033 | < 0.001 |
| Current | 1.127 (0.671, 1.582) | 0.057 | < 0.001 | -0.077 (-0.553, 0.400) | -0.004 | 0.753 |
| IADL score | 2.841 (2.767, 2.916) | 0.652 | < 0.001 | 1.733 (1.627, 1.838) | 0.398 | < 0.001 |
| BADL score | 0.456 (0.441, 0.471) | 0.564 | < 0.001 | 0.163 (0.145, 0.181) | 0.202 | < 0.001 |
| Leisure-time activity |  |  |  |  |  |  |
| light level（<= 115.75） | Reference |  |  | Reference |  |  |
| moderate（115.75~163.25） | 5.280 (4.750, 5.811) | 0.239 | < 0.001 | 1.247 (0.841, 1.652) | 0.056 | < 0.001 |
| vigorous（>163.25） | 6.054 (5,554, 6.555) | 0.291 | < 0.001 | 1.336 (0.932, 1.739) | 0.064 | < 0.001 |
| Household activity |  |  |  |  |  |  |
| light level（<= 8.60） | Reference |  |  | Reference |  |  |
| moderate（8.60~28.99） | 2.413 (1.889, 2.937) | 0.117 | < 0.001 | 1.539 （1.154, 1.925） | 0.075 | < 0.001 |
| vigorous（>28.99） | 4.442 (3.902, 4.983) | 0.209 | < 0.001 | 1.809 (1.406, 2.212) | 0.085 | < 0.001 |
| Work-related activity |  |  |  |  |  |  |
| No | Reference |  |  | Reference |  |  |
| Yes | 4.200 (3.479, 4.920) | 0.131 | < 0.001 | 0.756 （0.227, 1.286） | 0.024 | 0.005 |
| Depression | -11.300 (-11.918, -10.683) | -0.382 | < 0.001 | -2.046 （-2.584, -1.509） | -0.069 | < 0.001 |
| R2 |  |  |  | 0.503 |  |  |
| **Model 2: Dependent variable, MCS scores** |  |  |  |  |  |  |
| Age |  |  |  |  |  |  |
| 60-69 | Reference |  |  | Reference |  |  |
| 70-79 | -1.138 (-1.568, -0.708) | -0.061 | < 0.001 | 0.249 (-0.124, 0.621) | 0.013 | 0.190 |
| ≥80 | -4.083 (-4.633, -3.533) | -0.171 | < 0.001 | 0.326 (-0.234, 0.886) | 0.014 | 0.253 |
| Sex |  |  |  |  |  |  |
| Male | Reference |  |  |  |  |  |
| Female | -1.441 (-1.817, -1.065) | -0.086 | < 0.001 | -0.325 (-0.675, 0.025) | -0.019 | 0.069 |
| Education |  |  |  |  |  |  |
| Primary school or lower | Reference |  |  | Reference |  |  |
| Middle school or higher | 1.191 (0.809, 1.573) | 0.070 | < 0.001 | -1.073(-1.455, -0.691) | -0.063 | < 0.001 |
| Marital status |  |  |  |  |  |  |
| Married | Reference |  |  | Reference |  |  |
| Widowed/ divorced/ unmarried | -2.808 (-.324, -2.292) | -0.122 | < 0.001 | 0.254 (-0.293, 0.801) | 0.011 | 0.363 |
| Monthly income (Yuan) |  |  |  |  |  |  |
| ≤ 2000 | Reference |  |  | Reference |  |  |
| 2001–5000 | 2.186 (1.708, 2.664) | 0.127 | < 0.001 | 0.878 (0.444,1.312) | 0.051 | < 0.001 |
| ≥ 5001 | 3.916 (3.298, 4.534) | 0.176 | < 0.001 | 2.038 (1.448, 2.628) | 0.091 | < 0.001 |
| Living situation |  |  |  |  |  |  |
| Living with spouse/children | Reference |  |  | Reference |  |  |
| Living alone | -2.322 (-3.032, -1.611) | -0.074 | < 0.001 | -0.660 (-1.362, 0.041） | -0.021 | 0.065 |
| Smoking |  |  |  |  |  |  |
| Non-smoker | Reference |  |  | Reference |  |  |
| Former | 0.589 (-0.157, 1.336) | 0.018 | 0.122 | 0.034 (-0.693, 0.761) | 0.001 | 0.927 |
| Current | 1.490 (1.097, 1.882) | 0.088 | < 0.001 | -0.021 (-0.499, 0.465) | -0.001 | 0.930 |
| Alcohol drinking |  |  |  |  |  |  |
| Non-drinker | Reference |  |  | Reference |  |  |
| Former | -0.589 (-1.595, 0.417) | -0.013 | 0.251 | -0.134 (-1.077, 0.809) | -0.003 | 0.78 |
| Current | 1.598 (1.213, 1.983) | 0.095 | < 0.001 | 0.883 (0.411, 1.355) | 0.052 | < 0.001 |
| IADL score | 1.137 (1.057, 1.216) | 0.308 | < 0.001 | 0.154 (0.050, 0.259) | 0.042 | 0.004 |
| BADL score | 0.222 (0.207, 0.236) | 0.324 | < 0.001 | 0.065 (0.047, 0.082) | 0.094 | < 0.001 |
| Leisure-time activity |  |  |  |  |  |  |
| light level（<= 115.75） | Reference |  |  | Reference |  |  |
| moderate（115.75~163.25） | 1.942 (1.482, 2.401) | 0.104 | < 0.001 | 0.164（-0.237, 0.565） | 0.009 | 0.423 |
| vigorous（>163.25） | 3.766 (3.332, 4.201) | 0.213 | < 0.001 | 1.099（0.699, 1.499） | 0.062 | < 0.001 |
| Household activity |  |  |  |  |  |  |
| light level（<= 8.60） | Reference |  |  | Reference |  |  |
| moderate（8.60~28.99） | 1.466 (1.016, 1.916) | 0.084 | < 0.001 | 0.999 (0.617, 1.381) | 0.057 | < 0.001 |
| vigorous（>28.99） | 1.826 (1.362, .290) | 0.101 | < 0.001 | 0.598 (0.198, 0.997) | 0.033 | 0.003 |
| Work-related activity |  |  |  |  |  |  |
| No | Reference |  |  | Reference |  |  |
| Yes | 2.090 (1.476, 2.704) | 0.077 | < 0.001 | 0.429 (-0.096, 0.953) | 0.016 | < 0.001 |
| Depression | -13.533 (-14.010, -13.057) | -0.541 | < 0.001 | -11.657 (-12.190, -11.124) | -0.466 | < 0.001 |
